# Rural Aged Care Providers' Engagement in Medication Communication During Transitions of Care: A Qualitative Study

**DOI:** 10.1111/ajr.70161

**Published:** 2026-03-08

**Authors:** Alison Dowling, Elizabeth Manias

**Affiliations:** ^1^ School of Nursing and Midwifery Monash University Clayton Victoria Australia

**Keywords:** engagement, healthcare providers, medication communication, rural aged care, transitions of care

## Abstract

**Objective:**

To explore how healthcare providers in rural aged care homes communicate about medications during transitions of care involving residents.

**Setting:**

Two residential aged care homes in rural Australia.

**Participants:**

Ten female healthcare providers including nurses, personal care assistants and pharmacists.

**Design:**

Qualitative exploratory study using semi‐structured interviews, analysed thematically, with engagement mapped via the Patient and Family Engagement Framework. Consolidated Criteria for Reporting Qualitative Research guidelines were followed.

**Results:**

Two themes emerged from the interview data: (1) Providers' perceptions of medication communication roles and responsibilities, and (2) Providers' perceptions of challenges to engaging in medication communication. Nurses played a central but often reactive role, with resident and family engagement varying by capacity, proximity and interest. Personal care assistants and visiting pharmacists and personal care assistants contributed valuable safety and observational insights. Communication with other providers was fragmented and influenced by systemic and contextual inefficiencies.

**Conclusion:**

Healthcare providers in rural aged care homes, particularly nurses, face challenges in medication communication during care transitions. Structured, collaborative strategies are needed to ensure consistent information sharing and proactive resident and family engagement, with opportunities to strengthen contributions from pharmacists and personal care assistants in supporting person‐centred care.

## Abstract (highlights)

**What is already known on this subject?:**

- Residents in aged care homes frequently experience transitions of care, making them vulnerable to medication errors from communication breakdowns.
- Effective engagement and timely medication communication between healthcare providers, residents and families are critical but often suboptimal.
- Effective engagement and timely medication communication between healthcare providers, residents and families are critical but often suboptimal.
- Rural healthcare settings face unique challenges, including workforce shortages, geographic isolation, limited specialist access and resource constraints, which can hinder safe, coordinated medication communication.

**What this study adds?:**

- Offers insights into how rural aged care providers engage in medication communication during residents' transitions of care.
- Reveals barriers such as fragmented systems, inconsistent communication and workforce shortages that hinder engagement and medication safety.
- Suggests opportunities to improve communication through structured information‐sharing and collaborative, person‐centred strategies.

## 1 Introduction

Australia's ageing population, reflecting global trends, means older adults remain at home longer before entering residential aged care homes (RACHs) [1]. RACH residents are typically over 80 years, medically complex and often live with dementia or cognitive impairment [2]. Polypharmacy affects over two‐thirds of residents, with an average of 10.3 medicines daily [2], increasing the risk of medication errors, adverse drug events (ADEs), and hospital admissions [3]. Transitions of care, defined as movements between healthcare settings such as RACHs and hospitals, are high‐risk, particularly in rural contexts where systemic and geographic barriers add complexity [4]. Rural Australia sees approximately 72 500 medicine‐related hospital admissions annually, costing the healthcare system $400 million [5]. Contributing factors include fragmented services, poor information sharing, inconsistent documentation, limited pharmacy support, geographic isolation and long travel distances [6, 7].

Communication breakdowns during transitions of care among healthcare providers, RACH residents and families are major contributors to medication errors [8]. Poorly communicated medication changes can result in discrepancies, ADEs, readmissions, and preventable harm, with error rates of 16%–27% and ~17% of residents experiencing ADEs post‐discharge [9]. Residents typically undergo five to seven medication changes during hospitalisation [10], increasing the risk of overdosing, underdosing, inappropriate discontinuation or therapeutic duplication, potentially leading to functional decline, reduced quality of life, hospitalisation or death, and imposing substantial financial costs [11]. Improving medication communication is therefore critical. Engagement involves collaboration, shared decision‐making and mutual understanding, beyond simple information transfer [12]. Effective communication reduces discrepancies, supports safer medicine use and empowers residents and families, particularly in rural settings where risks are heightened [13].

Despite these concerns, research on medication communication during RACH transitions is limited, especially in rural contexts [8]. Existing studies have explored residents' and family caregivers' experiences [14] or provider perspectives in urban and rural settings [15], consistently showing suboptimal communication and limited engagement of residents and families. This highlights a critical evidence gap: little is known about how rural RACH healthcare providers engage residents, families and other providers in medication communication during transitions of care. Addressing this gap is essential to reducing preventable harm and supporting person‐centred care. Accordingly, this study aimed to explore how healthcare providers in rural Australian RACHs engage in medication communication during transitions of care.

## 2 Methods

### 2.1 Study Design

A qualitative exploratory design was used to investigate how rural RACH healthcare providers perceive and experience medication communication during care transitions with RACH residents and families and other healthcare providers [16]. This approach is suitable for exploring complex, context‐dependent issues in under‐researched settings and facilitates in‐depth understanding of participant perspectives. The study adhered to the Consolidated Criteria for Reporting Qualitative Research (COREQ) to ensure rigour and transparency [17] (See File S1). The Patient and Family Engagement Framework [12] guided data analysis, framing how healthcare providers engage residents and family members in medication communication during care transitions. The framework identifies three engagement levels; consultation (passive), involvement (active participation) and partnership/shared leadership (equal collaboration); allowing exploration of engagement depth in rural residential aged care. Although the framework primarily addresses healthcare provider–patient/family interactions, we extended its application to provider‐to‐provider communication, acknowledging its importance in supporting effective and coordinated medication management during care transitions. Table 1 presents an overview of the framework, adapted by the authors to align with the study's focus on healthcare provider engagement in medication communication during transitions of care involving rural RACH residents. The table outlines the three engagement levels and their application to both resident/family and provider‐to‐provider interactions.

**TABLE 1:** Overview of the patient and family engagement framework (adapted by study authors).

Engagement level	Description	Examples in the context of rural aged care medication communication
Consultation (passive)	Residents and families provide input or feedback, but providers make decisions independently.	Providers may ask residents/families about medication preferences but make changes without follow‐up or discussion.
Involvement (active participation)	Residents and families actively contribute to discussions about medications.	Participating in care planning meetings, asking questions about medication changes, or reviewing medication lists with nurses or pharmacists.
Partnership/Shared leadership (equal collaboration)	Residents, families, and healthcare providers share decision‐making responsibilities equally.	Co‐developing medication plans, jointly reviewing discharge medication instructions, collaborating with nurses, Personal Care Assistants (PCAs), and pharmacists to ensure safe transitions of care.

### 2.2 Setting

The study was conducted in two publicly funded rural RACHs in Australia (New South Wales and Victoria), both classified as MMM5 under the (deidentified) classification [18]. The (deidentified) is Australia's rurality classification system, developed by (deidentified) to define geographic remoteness and distribution of health services. MMM5 represents small rural towns with population under 5000, where limited health services and workforce shortages make them a particular focus for rural health policy and workforce incentives.

### 2.3 Participants

Healthcare providers involved in medication communication during transitions of care were recruited using purposeful sampling [19]. Participants included registered nurses (RNs), pharmacists and Personal Care Assistants (PCAs). The latter were included because in Australia, PCAs may assist with medication management in residential aged care homes, including tasks related to blister medication packs, provided they receive appropriate training and supervision [20]. Online promotion through professional networks, rural health organisations and Primary Health Networks broadened participation. Recruitment occurred between March and August 2024. Executive Directors and Regional Managers approved site participation, and nursing unit managers or RNs identified eligible participants. Author 1 contacted potential participants directly to provide study information and obtain written or verbal consent. Table 2 presents inclusion and exclusion criteria for RACH sites and participant groups.

**TABLE 2:** Study inclusion and exclusion criteria.

Criteria	Inclusion	Exclusion
*Rural residential aged care homes*		
Rural residential aged care homes	Situated in Victoria or southern New South Wales to minimise travel requirements for the study's data collector	RACHs located beyond a practical travel range for the study's data collector were excluded.
*Study participants*		
Healthcare providers	Have work experience in rural aged care homes, including roles such as follows: Registered/enrolled nurses Personal care assistants General Practitioners (GP) Pharmacists Allied health professionals AND Have direct involvement in transitions of care for aged care residents, where they participated in the communication of medication‐related information with: Other healthcare providers The transitioning resident The residents' family carer/s	Students are excluded from this project

### 2.4 Data Collection

Data were collected from 10 healthcare providers between June to August 2024 by Author 1 using a tailored semi‐structured interview guide and demographic questionnaire, adapted from validated tools on older adults' transitions of care [21]. Participants completed a brief demographic survey on age, gender and education, either on paper or via the Research Electronic Data Capture platform (REDCAP). Interviews were conducted in‐person at private rooms in rural aged care homes or remotely via Zoom, according to participant preference, and lasted on average 25 min (range 22–34). Open‐ended questions explored participants' roles in medication management, communication with residents, families and other healthcare providers, responsibilities for ensuring accurate information transfer, and essential medication information during transitions. For example:

- What is your role in managing residents' medications?
- How do you communicate with residents/family/other healthcare providers about residents' medication management?
  ○ Prompt: Could you walk me through how you usually share medication information with residents/family/other healthcare providers?
  ○ Prompt: Can you tell me about any challenges you face when doing so?
- What medication‐related information do you think should be shared during residents' transitions of care?
  ○ Prompt: Thinking about recent transitions of care, was there any information you felt was missing or would have been helpful to have?

Interviews were audio‐recorded, and in‐person participants received thank‐you cards and small gifts. Sampling continued until recurring themes emerged, guided by the concept of information power [22]. Information power considers the relevance and richness of data, the specificity of the sample and the quality of dialogue to determine when sufficient data have been collected [22]. In this study, it guided the sample size by ensuring the interviews provided adequate depth to answer the research question, rather than relying on a predetermined number of participants.

To make it clear who each quotation comes from while maintaining participant confidentiality, quotes are labelled according to the professional role of participants and allocated a unique number, indicating the order in which participants within each role were interviewed. For example, RN1 for Registered Nurse 1 (the first RN interviewed), PCA1 for Personal Care Assistant 1, Pharmacist1 for Pharmacist 1 and NUM1 for Nurse Unit Manager 1.

### 2.5 Data Analysis

Interviews were audio‐recorded and transcribed using Microsoft Word, with transcripts checked by Authors 1 and 2 for accuracy. Thematic analysis was conducted following Braun and Clarke's method [23]. See Table 3 for a summary of the Braun and Clarke process with examples from this study. The analysis process included familiarisation with the data, open coding of relevant text segments, grouping codes into broader categories and iterative refinement of themes. Initially, an inductive approach was applied to generate codes and identify themes directly from the data, allowing patterns and meanings to emerge without imposing preconceived categories. Subsequently, a deductive lens was used by mapping the emergent themes onto the Patient and Family Engagement Framework [12] to interpret levels of engagement in medication communication during care transitions. NVivo 14 software facilitated data management. Author 2 independently reviewed transcripts and coding decisions, and both authors finalised themes collaboratively to enhance rigour and credibility. Demographic data were analysed descriptively using Microsoft Excel to calculate frequencies, means and ranges.

**TABLE 3:** Thematic analysis process (Braun and Clarke [23])—Activities conducted in this study.

Steps (Braun and Clarke [23])	Description of activity in this study
1. Familiarisation with the data	Audio recordings were listened to, and transcripts were read and re‐read, with initial impressions noted.
2. Generating initial codes	Meaningful text segments related to medication communication, roles, responsibilities and engagement were open‐coded and organised systematically.
3. Searching for themes	Related codes were grouped into potential themes capturing patterns in how healthcare providers engaged in medication communication.
4. Reviewing themes	Themes were cross‐checked against the dataset to ensure they accurately reflected participant responses and the breadth of the data.
5. Defining and naming themes	Themes were refined, and clear definitions and labels were developed to capture the essence of each theme.
6. Producing the report	Final themes were synthesised into a narrative, supported with illustrative quotes and integrated into the manuscript.

### 2.6 AI‐Use Declaration

Grammarly AI was used to assist with clarity, readability, grammar, and structural review of text across the manuscript. It was not used to generate, draft or rewrite substantive content, nor to create ideas, interpretations, analyses or sections of the manuscript. All prompts involved checking or refining existing author‐written text. Grammarly AI outputs were reviewed, edited and verified by the authors for accuracy and appropriateness before inclusion. The authors take full responsibility for all content in the manuscript.

### 2.7 Research Team and Reflexivity

Author 1, a female Research Fellow with 5 years of qualitative research experience in aged care, conducted all interviews and data collection. She had no prior relationships with participants, which helped minimise potential influence on responses. Reflexivity was actively maintained by Author 1 through journaling after each interview to examine how Author 1's personal values, prior experiences, and professional identity could shape interactions and interpretation of data. Contextual factors, including the rural setting, workforce shortages and geographic isolation, were also considered during analysis. Author 2, a female Professor with clinical pharmacy and nursing qualifications, independently reviewed transcripts, coding and emerging themes. Regular supervisory discussions and team meetings further supported reflexivity, reduced bias and ensured that interpretations remained aligned with the study's aims.

## 3 Results

### 3.1 Characteristics of the Sample

Two government‐operated Australian rural RACHs participated in the study, contributing a total of 10 female healthcare provider participants. Two individuals who had given verbal consent to be contacted could not be reached and therefore did not participate.

The final sample included four registered nurses (RNs), two nursing unit managers (NUMs), two pharmacists and two personal care assistants (PCAs). Interviews were conducted either face‐to‐face (5 interviews) or via online platforms (5 interviews). See Table 4 for participant demographic details.

**TABLE 4:** Healthcare provider demographic information.

Characteristic	Healthcare providers, *n* (%)
*Participant characteristics*	
Age (years)	
Mean	45 years
Range	31–58 years
Gender	
Female	10 (100%)
Country of birth	
Australia	10 (100%)
Primary language	
English	10 (100%)
Highest education attained	
Diploma/certificate	2 (20%)
Bachelor's degree	7 (70%)
Master's degree	1 (10%)
Time working in RACH (months)	
Average	27
Range	4–60
Current work situation	
Full‐time (3 RNs, 2 NUMs, 2 PCAs)	7 (70%)
Part‐time (2 embedded pharmacists, working 1 day per week)	2 (20%)
Casual/Agency (1 RN)	1 (10%)
Current role in RACH	
Registered Nurse	4 (40%)
Nurse Unit Manager	2 (20%)
Embedded Pharmacist	2 (20%)
Personal Care Worker	2 (20%)

### 3.2 Themes

Two central themes were constructed through the analysis: [1] Providers' views on engagement in medication communication and [2] Providers' perceptions of challenges to engaging in medication communication, each with two sub‐themes. See Table 5 for a list of the themes. Note: For simplicity, the terms ‘Registered Nurses (RNs)’ and ‘Nurse Unit Managers (NUMs)’ are often combined as ‘nursing staff’ throughout to reflect their overlapping roles in medication management and communication.

**TABLE 5:** Themes derived from the data.

Themes
1.0 Providers' perceptions of medication communication roles and responsibilities
1.1 Providers' self‐perceived roles and responsibilities in medication communication
1.2 Providers' views on the medication communication roles of others
2.0 Providers' perceptions of challenges to engaging in medication communication
2.1 Differences in resident and family capacity and willingness to engage
2.2 System and context level influences on medication communication

Themes were initially identified inductively from participant interviews. These themes were then mapped against the Patient and Family Engagement Framework to provide a deductive lens. In this process, patterns identified in the interview data were considered in relation to the framework's levels of engagement: consultation, involvement and partnership/shared leadership (see Table 1). This placed participants' descriptions of medication communication and engagement within this framework.

#### 3.2.1 Providers' Perceptions of Medication Communication Roles and Responsibilities

This theme reflects participants' perceptions of their roles in medication communication during transitions of care. They explained that communication was influenced less by formal systems and more by challenges like rural workforce shortages, time constraints and fragmented processes. Participants described a mostly reactive approach to communication about medications; addressing issues and requests as they arose rather than following structured or proactive plans. As a result unclear responsibilities, inconsistent information flow and communication breakdowns with hospital staff and GPs often led to gaps that participants managed through ad hoc workarounds. Similarly, health care provider engagement with residents and family members tended to be reactive, shaped by assumptions, limited access and inconsistent processes. Altogether, these factors created a communication environment that was variable and sometimes unreliable across transitions of care.

##### 3.2.1.1 Providers' Self‐Perceived Roles and Responsibilities in Medication Communication

Healthcare providers in rural RACHs described roles and responsibilities encompassing education, administration, monitoring and liaison. Nurses were positioned at the centre of this communication, coordinating communication and compensating for gaps left by over‐stretched GPs:

It's us nursing staff who're primarily responsible for managing residents' medications here (in the RACH)…But we also work with GPs and pharmacists with us (nurses) at the centre. (RN5)

However, nurses also highlighted that limited GP availability in rural areas often required them to bridge communication gaps, highlighting how systemic constraints drove reactive communication practices:

If a resident or family member asks, I'm happy to provide information, but there isn't the time or staff for us to contact them and provide updates. (RN2)

Pharmacists described acting as a safety net, reconciling medications, monitoring use, and providing education to staff, residents, and families:

My role (at the RACH) is to do medication reviews to make sure the information is correct… I also monitor the use of medicines…and provide education for staff, residents and families. (Pharmacist2)

Pharmacists also led quarterly Medication Advisory Committee (MAC) meetings with GPs and nursing staff to review medication incidents, de‐prescribing, and other resident‐related matters.

PCAs, though not directly involved in medication discussions, contributed important observational insights due to their close relationships with residents and families that supported safe care:

We PCAs don't speak about medications…if a resident or family member asks I will go find the RN for them. We can dispense medications from webster packs, but only when we're short staffed. (PCA2)

Nursing staff recognised the value of PCAs' ongoing familiarity with residents, especially for temporary or agency nurses:

The PCAs are really good at monitoring the residents as they spend so much time with them… They know the residents and their families really well. (RN2)

Together, these findings show that while each healthcare provider group contribute to medication communication and safety, their actions are often shaped by limited staffing, unclear boundaries, and compensatory practices. Rather than operating within defined roles, communication relied on individuals steeping in to fill gaps created by systemic constraints.

##### 3.2.1.2 Providers' Views on the Medication Communication Roles of Others

Nursing staff reported that once residents were admitted to hospital, responsibility for medications shifted entirely to hospital teams, often with delayed or incomplete information returning to the RACH:

It becomes hospital's responsibility…We don't know anything until the resident gets back here…Even then the discharge documentation is often days late. I'm told they have up to four days to write it up. That to me is bad. (RN3)

Fragmented hospital‐RACH communication forced RACH staff to chase documentation to maintain resident safety. Nursing staff were aware that similar delays also affected local GPs:

Often they don't get anything (from the hospital) for days either so we can't always rely on them for clarity about medication changes made in the hospital. (RN1)

Communication challenges were heightened when residents were transferred to larger regional or metropolitan hospitals, where often due to staff turnover, siloed teams, and unclear roles created uncertainty about who to contact among RACH nursing staff:

…The discharge information from the bigger hospitals…can often turn up 3–4 days after the resident arrives back here…We can spend hours on the phone trying to track down the right doctor…Sometimes that doctor is not working that day or they are always not available to speak to you. (RN2)

By contrast, smaller rural hospitals or permanent local GPs enabled smoother communication about medications, reflecting the value of established relationships:

When we have a permanent GP in town, they work up at the hospital and here, so things run pretty smoothly…We can get verbal updates when the GP is here doing his rounds. (RN3)

Temporary or visiting GPs posed additional communication challenges because of their presence, requiring RACH staff to rely on telephone or email with no guarantee of timely responses:

When they come from the city, they're only in town for a few days…We just have to work around that and be ok with communicating by phone or email and know that you're probably not going to get an answer right away. (RN4)

Participants agreed that residents were often unable to take an active role in medication communication during transitions of care, particularly when unwell or cognitively impaired:

I don't think residents should be responsible. If they're going to hospital then they're usually pretty sick and probably not up to talking about their medications…Most of them don't know what they're taking so they can't be expected to talk about them. (RN1)

Family involvement, however, was seen as essential for ensuring residents' needs and preferences were understood and considered:

When residents come here it means they're no longer able to look after themselves and family are needed to speak on their behalf. That's important for the resident and for us. (RN2)

Overall, these findings highlight how systemic inefficiencies, inconsistent relationships across healthcare settings, and reliance on external providers influence medication communication. These challenges place a substantial burden on RACH staff, who must navigate unclear pathways while depending on when residents are unable to participate.

#### 3.2.2 Providers' Perceptions of Challenges to Engaging in Medication Communication

This theme highlights the range of systemic, relational, and contextual barriers that constrained healthcare providers' engagement in medication communication during transitions of care. Engagement was influenced by residents' cognitive capacity, health literacy, and willingness to participate, as well as by family involvement and the various communication systems between RACHS, hospitals, and GPs. Workforce instability in rural settings further disrupted continuity of care. Collectively, these challenges often resulted in healthcare providers having to rely on reactive, informal processes to main medication safety, as opposed to operating within defined responsibilities.

##### 3.2.2.1 Differences in Resident and Family Capacity and Willingness to Engage

Healthcare providers described wide variation in residents' and families' engagement, shaped by assumptions about interest, cognition, or confidence. Some residents and families preferred minimal information, influencing how nursing staff shared medication details:

I used to talk about medications to residents and the families, but some would say ‘I don't need to know all that.’ Now I just tell them what they ask for. (RN1)

Residents with cognitive impairment were less able to retain medication information, requiring repeated explanation, as explained by one nurse:

Some of the residents with dementia will ask all the time about their tablets, but they don't really understand or forget as soon as you tell them…But they will ask you again tomorrow. (RN4)

Other residents were described as being more engaged and proactive, seeking updates and detailed explanations about their medications, though they were in the minority:

Some residents want to know when things change and want a lot of detail. But it's not many. (RN1)

Family involvement was also varied, with closer or listed next of kin more engaged than distant family members. A NUM observed:

Those that live closer and visit regularly tend to ask more questions. It's those who live further away or aren't listed as next of kin that need to be encouraged to be more involved. (RN1)

Pharmacists faced additional challenges in communicating when residents did not recognise their role in medication management:

Since starting work here (at the RACH) some residents have said to me: ‘Why are you talking to me about my tablets? That's the doctor's role, not yours.’… Even now some still prefer talking only to the doctor. (Pharmacist2)

Several participants emphasised the value of conversational, open‐ended approaches to improve understanding and engagement. One PCA observed:

The way things are communicated could be better…by asking open‐ended questions…rather than just spilling out all this information that's just going over their head. (PCA1)

These findings highlight that engagement with residents and family members is highly variable, influenced by resident cognition, family preferences for involvement and role clarity.

##### 3.2.2.2 System and Context Level Influences on Medication Communication

RACH staff described how fragmented communication systems and inefficient processes increased their workload and limited effective and timely communication. For example, duplicated documentation and repeated requests from hospitals created additional burden on RACH nursing staff:

When we send someone to hospital, we print and send everything…care plans, drug chart, progress notes…Then the hospital will ring and ask for the information we already sent…It wastes a lot of time. (NUM2)

Geographic isolation and transfers to larger regional and metropolitan hospitals increased communication challenges for nursing staff:

Things can get messy when residents go to the bigger hospitals…There's no standardised system for sharing information…We just have to hope the resident brings the discharge documentation, which doesn't always happen. (NUM1)

By contrast, familiarity with smaller rural hospitals facilitated smoother communication:

Between here and the local (rural) hospital, it's usually fine…We all know one another so we can ring and get the information we need. At the bigger hospitals, it's potluck who you talk to. (NUM1)

Workforce instability, including transient doctors and casual nursing staff disrupted continuity and resident familiarity:

…Our doctors are fly in, fly out…They seem to change every week and we have to go over resident histories over and over…They write scripts for residents they don't know, which has resulted in some bad side effects. (NUM2)

An agency nurse further highlighted the challenges of workforce casualisation:

I travel up from the city to do 3‐5 shifts a month…Sometimes residents ask me to get one of the permanent RNs because they don't remember who I am. (RN4)

Overall, these findings show that staffing challenges in rural areas, inconsistent documentation, and fragmented communication systems make it difficult for healthcare providers to engage proactively with residents and families. As a result, providers often have to rely on reactive approaches, drawing on their own knowledge and relationships, rather than benefiting from consistent, system wide processes.

## 4 Discussion

This is the first known Australian study to examine how healthcare providers in rural RACHs communicate about medications during care transitions. Nursing staff were central coordinators, linking residents, families, GPs and pharmacists, yet communication was often reactive rather than structured. Continuity was disrupted by systemic inefficiencies, fragmented hospital–RACH communication, geographic isolation and workforce shortages, with staff frequently chasing delayed or incomplete documentation. Engagement with residents and families varied according to cognitive capacity, proximity and interest. Pharmacists and PCAs contributed important safety and observational insights, and the findings suggest their roles could be expanded to strengthen medication communication in rural RACHs. Together, these findings point to the need for clearer role delineation, more systematic and proactive information‐sharing processes, and strengthened interprofessional collaboration to support safer, person‐centred medication communication in rural aged care.

This study found that healthcare providers in rural RACHs, particularly nurses, often engaged residents and family members reactively rather than proactively in medication communication, reflecting assumptions about preferences and shaped by systemic constraints. Similar patterns of reactive nurse‐led communication have been reported elsewhere, limiting opportunities for shared decision‐making [8]. In rural settings, workforce shortages, transient staff and fragmented systems further disrupt timely collaboration among providers, residents, and families [24]. While participants perceived limited resident and family interest, other studies suggest many value active involvement [8]. At the same time, medication complexity and low health literacy, particularly in rural populations, may contribute to reliance on healthcare providers [25]. These mixed findings underscore the need to assess individual preferences and tailor engagement strategies. Supporting residents and families to participate more actively in medication discussions can strengthen understanding, confidence and decision‐making [26], which is particularly critical in rural contexts with limited access to information and support [6].

Workforce shortages also constrained RACH providers' engagement with external providers during transitions of care. Communication with GPs and other providers, many without a permanent rural presence, often relied on phone or email, contributing to fragmented care. High turnover and transient staff disrupted continuity, as providers frequently lacked knowledge of residents' histories and preferences [27]. Disorganised processes further impeded teamwork and trust, undermining person‐centred care and increasing risks of medication errors [28]. Persistent recruitment and retention challenges continue to strain workloads and limit time for resident and family engagement [29]. Systemic workforce challenges, such as rural healthcare teams being almost 60% smaller than their metropolitan counterparts, reflect global patterns of rural maldistribution [30]. Despite government strategies, such as recruiting overseas‐trained doctors, most remain in metropolitan areas [31]. Addressing these entrenched issues requires investment in workforce stability, interprofessional collaboration and integrated communication systems.

The findings also highlight the potential value of expanding the roles of pharmacists and PCAs to support medication management during RACH care transitions. Pharmacists, including those in hospitals where breakdowns with RACH providers often occur, currently provide part‐time, non‐mandated services in Australia [32]. Expanding their role could strengthen medication safety by improving communication, reconciliation, and education for residents, family members and healthcare providers; especially in rural areas facing workforce shortages and geographic isolation [6]. For example, on‐site pharmacists embedded part‐time in RACH care teams have been shown to reduce the use of potentially harmful medications and improve medication appropriateness [33]. Research consistently shows pharmacist‐led strategies, delivered collaboratively with other healthcare providers, are most effective during transitions of care [34]. This study also highlights the often under‐recognised role of PCAs in medication‐related communication [35]. Through their close, ongoing contact with residents, PCAs are well placed to notice subtle health changes and support nursing staff and other healthcare providers, via informal communication. They provide about 70% of direct care and comprise 60%–80% of the aged care workforce [32], positioning them as key contributors to safety and communication. However, their potential impact is limited by training and role boundaries [35]. In the context of persistent nursing shortages in rural aged care, targeted training and clearer role definitions could strengthen their contributions and support person‐centred, multidisciplinary care [13].

Application of the Patient and Family Engagement Framework [12] revealed that engagement in medication communication in rural aged care homes occurred at multiple levels. However, residents' involvement was generally limited, particularly for those with cognitive impairment or acute illness, and mostly occurred at the consultation level, with minimal influence on decisions. Family members often acted as intermediaries when residents could not advocate for themselves, while workforce shortages and variable family participation constrained deeper partnership or shared decision‐making. Engagement between RACH staff and external providers, such as hospitals and GPs, ranged from reactive information sharing; chasing delayed discharge summaries or clarifications from temporary GPs; to more proactive coordination where established relationships existed, such as with permanent local GPs or smaller rural hospitals. Fragmented communication, workforce turnover and geographic isolation limited effective care coordination, highlighting systemic barriers. These findings align with other studies, which found that rural residents and family caregivers experienced primarily consultative communication with minimal proactive engagement, and that residents' and caregivers' perspectives were often under‐addressed due to systemic and relational barriers [8, 14]. Overall, mapping the findings revealed gaps in partnership and shared decision‐making. Staff maintained medication safety, but resident and family engagement rarely moved beyond consultation. The framework highlights opportunities to strengthen active engagement through structured communication, stable staffing, and consistent family involvement, supporting person‐centred care and medication safety.

## 5 Conclusion

The findings indicate that healthcare providers in rural RACHs often engage residents and family members reactively rather than proactively in medication decisions, while collaboration with external providers is constrained by fragmented systems, inconsistent communication, and limited access to GPs and hospital staff. These results underscore the need for systematic information‐sharing processes and a shift from passive information provision toward genuine partnership. Persistent workforce instability further challenges safe, coordinated care, highlighting the importance of stabilising the rural healthcare workforce. Expanding the roles of PCAs and pharmacists through targeted training, clearer responsibilities and strengthened collaboration could enhance communication, continuity and person‐centred medication management. Collectively, these findings offer preliminary evidence to inform policy, guide practice reforms and direct future research aimed at improving engagement, safety and outcomes in rural aged care.

## 6 Limitations and Strengths

This study has several limitations. Recruitment was limited to two rural RACHs, with expansion constrained by staffing shortages and COVID‐19 outbreaks. The research team's geographical distance required reliance on RACH staff for recruitment and distribution of materials, which may have introduced bias or inconsistencies. Additionally, the small sample size and focus on rural settings may limit transferability to metropolitan or international contexts. Despite these limitations, the study provides valuable insights from healthcare providers in rural RACHs, offering important baseline evidence on medication communication. Its integrated perspective is novel in the Australian context and informs strategies to enhance safety and collaboration.

## 7 Implications for Policy and Practice

Nurses in rural RACHs are central to medication communication during care transitions and require support to lead improvements. Short‐term strategies include assessing individual preferences using tools such as RESPECT [36] and PELI‐NH [37] to guide person‐centred engagement, along with training in plain language, teach‐back and adaptive communication techniques [38]. Embedding structured communication tools like ISBAR [39] and TeamSTEPPS [40] into workflows can strengthen interprofessional coordination, while proactive strategies such as care conferences and medication summaries support continuity and trust. Digital platforms (e.g., MedAdvisor, Healthdirect and MedicineWise) may enhance medication management but require training and infrastructure. Longer term strategies requiring policy, education and government support include increasing pharmacists' presence for medication reconciliation, expanding communication options such as telehealth with staff training and training PCAs to support medication communication, reducing nursing workload and enhancing safety. Recognising nurses as key leaders is essential to implementing these strategies and strengthening person‐centred medication communication in rural aged care.

## Author Contributions

**Alison Dowling:** conceptualization, writing – original draft, writing – review and editing, methodology, software, formal analysis, project administration, data curation, supervision, investigation. **Elizabeth Manias:** writing – review and editing, supervision, methodology.

## Disclosure

The authors received no financial support for the research, authorship and/or publication of this manuscript. The authors alone are responsible for the content and writing of the manuscript.

## Ethics Statement

This study received ethical approval from the Monash University Human Research Ethics Committee (Project ID: 41401). Participation was voluntary, with written consent obtained before interviews. Participants could withdraw at any time, and confidentiality was maintained.

## Conflicts of Interest

The authors declare no conflicts of interest.

## Supporting information


**File S1:** ajr70161‐sup‐0001‐AppendixS1.docx.

## Data Availability

The dataset generated during this study are not publicly available because participants did not provide consent for their data to be shared. Researchers seeking to discuss the findings are encouraged to contact the corresponding author for further information, within the constraints of participant confidentiality.

## References

[1] R. Elliott and J. Booth , “Problems With Medicine Use in Older Australians: A Review of Recent Literature,” Journal of Pharmacy Practice and Research 44, no. 4 (2014): 258–271.

[2] A. Page , K. Potter , V. Naganathan , et al., “Polypharmacy and Medicine Regimens in Older Adults in Residential Aged Care,” Archives of Gerontology and Geriatrics 105 (2023): 104849. 36399891 10.1016/j.archger.2022.104849

[3] R. Desai , C. Williams , S. Greene , S. Pierson , and R. Hansen , “Medication Errors During Patient Transitions Into Nursing Homes: Characteristics and Associations With Patient Harm,” American Journal of Geriatric Pharmacotherapy 9, no. 6 (2011): 413–422. 22078862 10.1016/j.amjopharm.2011.10.005

[4] J. Penm , K. Yeung , R. Moles , et al., “Timely Post‐Discharge Medication Reviews to Improve Continuity—The Transitions of Care Stewardship (TIC TOC) Study in Rural and Regional Australia: A Parallel‐Group Randomised Controlled Trial Study Protocol,” BMJ Open 15, no. 6 (2025): e100588. 10.1136/bmjopen-2025-100588 PMC12182129 40533208

[5] Pharmaceutical Society of Australia , Medicine Safety (Rural and Remote Care, 2021).

[6] D. Piper , V. Parker , and J. Gray , “Communicating Across Rural and Metropolitan Health Care Settings,” in Communicating Quality and Safety in Health Care, ed. R. Iedema , D. Piper , and M. Manidis (Cambridge University Press, 2015), 49–65. 2015.

[7] D. Piper , J. Lea , C. Woods , and V. Parker , “The Impact of Patient Safety Culture on Handover in Rural Health Facilities,” BMC Health Services Research 18, no. 1 (2018): 889–913. 30477488 10.1186/s12913-018-3708-3 PMC6257960

[8] A. Dowling , D. Garratt , and E. Manias , “Experiences and Perceptions of Medication Management Communication During Transitions of Care for Residents in Aged Care Homes and Their Caregivers: A Qualitative Meta‐Synthesis,” Journal of Clinical Nursing 34 (2024): 1–20. 10.1111/jocn.17438 PMC11933520 39370545

[9] N. Ferrah , J. Lovell , and J. Ibrahim , “Systematic Review of the Prevalence of Medication Errors Resulting in Hospitalization and Death of Nursing Home Residents,” Journal of the American Geriatrics Society 65, no. 2 (2017): 433–442. 27870068 10.1111/jgs.14683

[10] R. A. Elliott , “Problems With Medication Use in the Elderly: An Australian Perspective,” Journal of Pharmacy Practice and Research 36, no. 1 (2006): 58–66.

[11] R. A. Elliott , Y. Boutros , T. Tran , and S. E. Taylor , “A Prospective Study of Medication Management During Transitions From Hospital to Residential Care: A 10‐Year Follow‐Up to the MedGap Study,” Journal of Pharmacy Practice and Research 50, no. 4 (2020): 308–315.

[12] K. Carman , P. Dardess , M. Maurer , et al., “Patient and Family Engagement: A Framework for Understanding the Elements and Developing Interventions and Policies,” Health Affairs 32, no. 2 (2013): 223–231. 23381514 10.1377/hlthaff.2012.1133

[13] X. Zhou , F. Du , W. Peng , L. Bai , L. Peng , and X. Hou , “Building Medication Profiles in the Elderly: A Qualitative Study Based on Medication Information Literacy in a Long‐Term Care Facility,” Clinical Interventions in Aging 19 (2024): 779–793. 38751855 10.2147/CIA.S454620 PMC11095403

[14] A. Dowling and E. Manias , “Engagement in Medication Communication During Transitions of Care for Rural Aged Care Residents and Family Caregivers: A Qualitative Study,” Journal of Clinical Nursing 35 (2025): 850. 40709527 10.1111/jocn.70047 PMC12779257

[15] A. Pulst , A. Fassmer , F. Hoffmann , and G. Schmiemann , “Paramedics' Perspectives on the Hospital Transfers of Nursing Home Residents‐A Qualitative Focus Group Study,” International Journal of Environmental Research and Public Health 17, no. 11 (2020): 1–16. 10.3390/ijerph17113778 PMC7312002 32466568

[16] J. Creswell and J. Creswell , Research Design: Qualitative, Quantitative, and Mixed Methods Approaches, 5th ed. (SAGE Publications, Inc., 2018).

[17] A. Tong , P. Sainsbury , and J. Craig , “Consolidated Criteria for Reporting Qualitative Research (COREQ): A 32‐Item Checklist for Interviews and Focus Groups,” International Journal for Quality in Health Care 19, no. 6 (2007): 349–357. 17872937 10.1093/intqhc/mzm042

[18] Australian Government Department of H, Aged C , Modified Monash Model (Australian Government, 2024).

[19] L. Palinkas , S. Horwitz , C. Green , J. Wisdom , N. Duan , and K. Hoagwood , “Purposeful Sampling for Qualitative Data Collection and Analysis in Mixed Method Implementation Research,” Administration and Policy in Mental Health and Mental Health Services Research 42, no. 5 (2015): 533–544. 24193818 10.1007/s10488-013-0528-y PMC4012002

[20] Federation ANaM , Best Practice Guidance for Medicines Use by Nurses in Aged Care (Federation ANaM, 2025).

[21] E. Manias , S. Rixon , A. Williams , D. Liew , and S. Braaf , “Barriers and Enablers Affecting Patient Engagement in Managing Medications Within Specialty Hospital Settings,” Health Expectations 18, no. 6 (2015): 2787–2798. 25186633 10.1111/hex.12255 PMC5810626

[22] K. Malterud , V. Siersma , and A. Guassora , “Sample Size in Qualitative Interview Studies: Guided by Information Power,” Qualitative Health Research 26, no. 13 (2016): 1753–1760. 26613970 10.1177/1049732315617444

[23] V. Clarke and V. Braun , “Using Thematic Analysis in Counselling and Psychotherapy Research: A Critical Reflection,” Counselling and Psychotherapy Research 18, no. 2 (2018): 107–110.

[24] National Rural Health Alliance , Aged Care Access in Rural Australia (National Rural Health Alliance, 2022).

[25] N. Coombs , D. Campbell , and J. Caringi , “A Qualitative Study of Rural Healthcare Providers' Views of Social, Cultural, and Programmatic Barriers to Healthcare Access,” BMC Health Services Research 22, no. 1 (2022): 438. 35366860 10.1186/s12913-022-07829-2 PMC8976509

[26] M. Sawan , Y. Jeon , S. Hilmer , and T. Chen , “Perspectives of Residents on Shared Decision Making in Medication Management: A Qualitative Study,” International Psychogeriatrics 34, no. 10 (2022): 929–939. 35357282 10.1017/S1041610222000205

[27] N. Ma , N. Sutton , J. Yang , et al., “The Quality Effects of Agency Staffing in Residential Aged Care,” Australasian Journal on Ageing 42, no. 1 (2023): 195–203. 35997130 10.1111/ajag.13132

[28] I. Winqvist , U. Näppä , and M. Häggström , “Quality of Care During Rural Care Transitions: A Qualitative Study on Structural Conditions,” BMC Nursing 22, no. 1 (2023): 1–14. 37559083 10.1186/s12912-023-01423-5 PMC10411022

[29] A. Chapman , A. Buccheri , D. Mohotti , et al., “Staff‐Reported Barriers and Facilitators to the Implementation of Healthcare Interventions Within Regional and Rural Areas: A Rapid Review,” BMC Health Services Research 25, no. 1 (2025): 1–18. 40033247 10.1186/s12913-025-12480-8 PMC11877690

[30] I. Blackberry and N. Morris , “The Impact of Population Ageing on Rural Aged Care Needs in Australia: Identifying Projected Gaps in Service Provision by 2032,” Geriatrics 8, no. 3 (2023): 47. 37218827 10.3390/geriatrics8030047 PMC10204523

[31] C. Cortie , D. Garne , L. Parker‐Newlyn , et al., “The Australian Health Workforce: Disproportionate Shortfalls in Small Rural Towns,” Australian Journal of Rural Health 32, no. 3 (2024): 538–546. 38597124 10.1111/ajr.13121

[32] Australian Government Department of Health and Aged Care , 2020 Aged Care Workforce Census Report. Ageing and Aged Care for Workforce (Australian Government, 2020).

[33] I. Haider , S. Kosari , M. Naunton , et al., “Impact of On‐Site Pharmacists in Residential Aged Care Facilities on the Quality of Medicines Use: A Cluster Randomised Controlled Trial (PiRACF Study),” Scientific Reports 13, no. 1 (2023): 15962. 37749102 10.1038/s41598-023-42894-5 PMC10519995

[34] F. Emadi , S. Liu , C. Yiu , et al., Strategies to Facilitate Safer Medication Management at Transitions of Care. Evidence Briefings on Interventions Supporting Safer Transitions of Care (Australian Commission on Safety and Quality in Health Care, 2024).

[35] Australian College of Nursing , Regulation of the Unregulated Health Care Workforce Across the Health Care System – A White Paper (Australian College of Nursing, 2019).

[36] A. Damiaens , A. Van Hecke , and V. Foulon , “The RESPECT‐Tool as a Facilitator for Person‐Centered Medication Reviews for Nursing Home Residents: Tool Development and Pilot Study,” International Journal of Clinical Pharmacy 45, no. 6 (2023): 1434–1443. 37493905 10.1007/s11096-023-01621-w

[37] K. Curyto , K. Van Haitsma , and G. Towsley , “Revising the Preferences for Everyday Living Inventory for Use in the Nursing Home,” Research in Gerontological Nursing 9, no. 1 (2015): 24–34. 26020577 10.3928/19404921-20150522-04 PMC7990027

[38] P. Yen and A. R. Leasure , “Use and Effectiveness of the Teach‐Back Method in Patient Education and Health Outcomes,” Federal Practitioner 36, no. 6 (2019): 284–289. 31258322 PMC6590951

[39] A. Burgess , C. van Diggele , C. Roberts , and C. Mellis , “Teaching Clinical Handover With ISBAR,” BMC Medical Education 20, no. Suppl 2 (2020): 459. 33272274 10.1186/s12909-020-02285-0 PMC7712559

[40] C. McCaskill , “TeamSTEPPS: The Introduction of Leadership, Situational Monitoring, Mutual Support and Communication to Improve Patient Safety in an Emergency Department,” Australasian Emergency Nursing Journal 14 (2011): S44.

