# Supplementary material for: Rural Aged Care Providers' Engagement in Medication Communication During Transitions of Care: A Qualitative Study
Source: Aust J Rural Health. 2026 Mar 8;34(2):e70161. doi: 10.1111/ajr.70161 (PMC12968489; doi:10.1111/ajr.70161)
Supplement: Supplementary file 1 — File S1: ajr70161‐sup‐0001‐AppendixS1.docx. [file AJR-34-0-s001.docx]

**Supplementary file 1**

Consolidated criteria for reporting qualitative studies (COREQ): 32-item checklist

| **No** | **Item** | **Guide questions/description** | **Page no in manuscript/comment** |
| --- | --- | --- | --- |
| **Domain 1: Research team and reflexivity** |  |  |  |
| Personal Characteristics |  |  |  |
| 1. | Interviewer/facilitator | Which author/s conducted the interview or focus group? | P5 (Data Collection) |
| 2. | Credentials | What were the researcher's credentials? *E.g. PhD, MD* | P6 (Rigour and reflexivity) |
| 3. | Occupation | What was their occupation at the time of the study? | P6 (Rigour and reflexivity) |
| 4. | Gender | Was the researcher male or female? | P6 (Rigour and reflexivity) |
| 5. | Experience and training | What experience or training did the researcher have? | P6 (Rigour and reflexivity) |
| Relationship with participants |  |  |  |
| 6. | Relationship established | Was a relationship established prior to study commencement? | P6 (Rigour and reflexivity)  No relationship was established. |
| 7. | Participant knowledge of the interviewer | What did the participants know about the researcher? e*.g. personal goals, reasons for doing the research* | The participants did not know any of the researchers. However, all participants knew that the interview was for research purposes. |
| 8. | Interviewer characteristics | What characteristics were reported about the interviewer/facilitator? e.g. *Bias, assumptions, reasons and interests in the research topic* | None.  Participants were introduced to the research using the ethics approved Explanatory Statement and Consent Form. |
| **Domain 2: study design** |  |  |  |
| Theoretical framework |  |  |  |
| 9. | Methodological orientation and Theory | Exploratory descriptive qualitative approach was used to gather in-depth insights into the topic of interest (medication management communication during transitions of care) by exploring it from the perspective of participants, without the constraint of predefined hypotheses. In this type of study, the goal was to explore and describe the experiences and perceptions of participants. Thematic analysis was used to analyze the study data. | P1 (Abstract)  P3 (Study Design) |
| Participant selection |  |  |  |
| 10. | Sampling | How were participants selected? *e.g. purposive, convenience, consecutive, snowball* | p1 (Method)  p4 (Sampling)  Purposive sampling was used |
| 11. | Method of approach | How were participants approached? e*.g. face-to-face, telephone, mail, email* | Various methods of approach were used. These are explained under P4-5 |
| 12. | Sample size | How many participants were in the study? | p1 (Method)  p5 (Data Collection)  p7 (Characteristics of the sample) |
| 13. | Non-participation | How many people refused to participate or dropped out? Reasons? | P7 (Characteristics of the sample)  Two individuals who gave verbal consent to be contacted by Author 1 could not be reached and did not participate in the interviews. |
| Setting |  |  |  |
| 14. | Setting of data collection | Where was the data collected? e*.g. home, clinic, workplace* | P5 (Data collection) |
| 15. | Presence of non-participants | Was anyone else present besides the participants and researchers? | Nil, not relevant. |
| 16. | Description of sample | What are the important characteristics of the sample? *e.g. demographic data, date* | Described in Table 1 and p6-7 (Characteristics of the sample) |
| Data collection |  |  |  |
| 17. | Interview guide | Were questions, prompts, guides provided by the authors? Was it pilot tested? | Interview guide and demographic questionnaire were developed and used. These are explained under on P5 (Participants) |
| 18. | Repeat interviews | Were repeat interviews carried out? If yes, how many? | No repeat interviews were carried out as this was not relevant to the study aims or study rationale |
| 19. | Audio/visual recording | Did the research use audio or visual recording to collect the data? | Yes, noted on p5 (Data collection and Data analysis) |
| 20. | Field notes | Were field notes made during and/or after the interview or focus group? | P6 (Research team & reflexivity). Author 1 journaled reflections on how personal beliefs, values, and aged care experience might influence interpretation, including emotional tone and interviewer-participant dynamics. |
| 21. | Duration | What was the duration of the interviews? | P5 (Data collection) Interviews averaged 25 minutes in length (range: 22–34 minutes). |
| 22. | Data saturation | Was data saturation discussed? | No, not applicable due to the number of participants – Data collection in this qualitative study was guided by the concept of information power (p5) |
| 23. | Transcripts returned | Were transcripts returned to participants for comment and/or correction? | No member checks were carried out as this was not relevant to the study aims or rationale for the study. Nor was it practical. |
| **Domain 3: analysis and findings**z |  |  |  |
| Data analysis |  |  |  |
| 24. | Number of data coders | How many data coders coded the data? | P5 (Data analysis) |
| 25. | Description of the coding tree | Did authors provide a description of the coding tree? | No |
| 26. | Derivation of themes | Were themes identified in advance or derived from the data? | Themes were derived from the data (p5 – Data analysis) |
| 27. | Software | What software, if applicable, was used to manage the data? | NVivo software was used (p6) |
| 28. | Participant checking | Did participants provide feedback on the findings? | Not applicable. |
| Reporting |  |  |  |
| 29. | Quotations presented | Were participant quotations presented to illustrate the themes / findings? Was each quotation identified? e*.g. participant number* | Quotations provided under Results section.  (pg8-13) |
| 30. | Data and findings consistent | Was there consistency between the data presented and the findings? | Yes, quotes were provided verbatim. Quotations provided under Results section |
| 31. | Clarity of major themes | Were major themes clearly presented in the findings? | Yes (p7) and Table 5 |
| 32. | Clarity of minor themes | Is there a description of diverse cases or discussion of minor themes? | Minor themes provided in Results section (pg8-13) |
